# Combination of inductive effect of lipopolysaccharide and in situ mechanical conditioning for forming an autologous vascular graft in vivo

**DOI:** 10.1038/s41598-019-47054-2

**Published:** 2019-07-23

**Authors:** Chao-Lin Chen, How-Ran Guo, Ying-Jan Wang, Hong-Tai Chang, Chui-Yi Pan, Ho-Yi Tuan-Mu, Hsiu-Chuan Lin, Chao-Yi Chen, Jin-Jia Hu

**Affiliations:** 1 Department of Biomedical Engineering, National Cheng Kung University, Tainan, Taiwan; 2 Department of Occupational Medicine, Kaohsiung Veterans General Hospital, Kaohsiung, Taiwan; 3 Department of Environmental and Occupational Health, College of Medicine, National Cheng Kung University, Tainan, Taiwan; 4 Department of Occupational and Environmental Medicine, National Cheng Kung University Hospital, Tainan, Taiwan; 5 Division of General Surgery, Department of Surgery, Kaohsiung Veterans General Hospital, Kaohsiung, Taiwan; 6 Chest Hospital, Ministry of Health and Welfare, Tainan, Taiwan; 7 Department of Physical Therapy, Tzu Chi University, Hualien, Taiwan; 8 Medical Device Innovation Center, National Cheng Kung University, Tainan, Taiwan

**Keywords:** Regenerative medicine, Tissue engineering

## Abstract

Autologous vascular grafts have the advantages of better biocompatibility and prognosis. However, previous studies that implanted bare polymer tubes in animals to grow autologous tubular tissues were limited by their poor yield rates and stability. To enhance the yield rate of the tubular tissue, we employed a design with the addition of overlaid autologous whole blood scaffold containing lipopolysaccharides (LPS). Furthermore, we applied *in vivo* dynamic mechanical stimuli through cyclically inflatable silicone tube to improve the mechanical properties of the harvested tissues. The effectiveness of the modification was examined by implanting the tubes in the peritoneal cavity of rats. A group without mechanical stimuli served as the controls. After 24 days of culture including 16 days of cyclic mechanical stimuli, we harvested the tubular tissue forming on the silicone tube for analysis or further autologous interposition vascular grafting. In comparison with those without cyclic dynamic stimuli, tubular tissues with this treatment during *in vivo* culture had stronger mechanical properties, better smooth muscle differentiation, and more collagen and elastin expression by the end of incubation period in the peritoneal cavity. The grafts remained patent after 4 months of implantation and showed the presence of endothelial and smooth muscle cells. This model shows a new prospect for vascular tissue engineering.

## Introduction

Vascular grafts are needed for many clinical conditions such as coronary artery disease, end-stage renal disease, and peripheral artery occlusion disease. Many of these conditions are prevalent and critical. For example, coronary artery disease is one of the major causes of mortality and morbidity around the world. In the U.S., it was estimated that 660,000 new coronary events (first hospitalized myocardial infarction or coronary artery disease death), and about 305,000 recurrent coronary events would take place in one year^1^. In 2010, it was estimated that 397,000 inpatient bypass procedures were performed^1^.

In such procedures, the choice of vascular grafts is critical. For example, among the coronary artery bypass grafting procedures, the re-do procedures have worse outcomes than first-performed procedures^2,3^. Radial arteries are more often used in re-do procedures^2^, but result in a lower patency rate than left internal mammary artery and saphenous vein grafts^4^. The lack of suitable bypass grafts is still a main problem during re-do bypass procedures and may be related to worse outcomes compared with first-performed bypass^5^. However, there is still no adequate prosthetic conduit for coronary artery bypass grafting^6^.

Tissue engineering offers hope for vascular bypass grafting. Although there has been a clinical trial on tissue engineered blood vessels (TEBV) composed of autologous cells without synthetic materials, the long production time (7.5 months on average) hampers its application^7^. The other way to develop TEBV with autologous cells is to cultivate them *in vivo*, i.e. in the body of the receiver. In the clinical trial conducted by Sparks *et al*., the use of autologous subcutaneous tissue as vascular grafts resulted in unfavorable outcomes. However, the grafts contained synthetic Dacron mesh, a non-degradable foreign body, for the purpose of better mechanical support^8^. For 100% autologous subcutaneous tissue tubes, methods had been proposed for making them stronger^9–11^, including *in vivo* cyclic mechanical stimulation^12^, which has been well documented in the field of *in vitro* fabrication of TEBV^13–15^.

Some researchers have tried to use the peritoneal cavity to cultivate vascular grafts through forming autologous tubular tissue around implanted silicone tubes, and the anti-coagulation property of the inverted mesothelium fits the characteristic of the luminal side (Campbell *et al*.^16^). But the model is limited by the yield rate of the tubular tissue, ranging from one to three out of four^16–18^. While adding polymer sheets or meshes could achieve a 100% yield rate^18^, the tubular tissue is not completely autologous. This method had also been applied to the autologous transplantation of organs such as the bladder, uterus, and vas deferens (use polyethylene balls and boiled blood clots as molds)^19^, as well as urethral grafts (use silicone tubes as molds)^17^, and yielded favorable outcomes. This proves its utility in regenerative medicine. However, the yield rates were not detailed in the reports, and the reported durations that were needed to produce useful grafts in mouse models ranged from 2 to 8 weeks^16–18,20^. This limitation might add uncertainty to its further application. It is known that plasma treatment combined with collagen coating on the silicone material can optimize biocompatibility^21^, but the stability of pre-coated collagen is unpredictable *in vivo*. Since collagen can be synthesized through activating macrophages, we used lipopolysaccharide (LPS), which is a potent macrophage activator, as a “booster” to yield autologous and applicable extracellular matrix (ECM) within receiver’s body. Here we introduce an *in vivo* tissue-engineering system using a rodent animal model, which includes the application of LPS, autologous whole blood scaffold, and cyclic mechanical stimulation in the “bioreactor” of the peritoneal cavity. From the peritoneal cavity, the vascular graft is fabricated and implanted as an autologous interpositional aortic graft for up to 4 months. We hereby conducted a study to determine the mechanical properties, patency rate, ECM expression, and smooth muscle differentiation of the vascular grafts produced through this method.

## Materials and Methods

### Inflatable silicone tubes

Two sizes of ultra-soft silicone tubes were custom-ordered (Dicheng Rubber Industries Inc., Taiwan) for our experiment. The 2.5-mm tubes were used for generating tubular tissues and subsequent autologous grafts as aortic interposition, with a 17.65% increment of external diameter from 0 to 3.5 kg/cm^2^ pressurization. The 2.75-mm tubes were used for generating tubular tissues with subsequent mechanical testing and protein/ECM (Western blot and collagen/elastin) analysis on the harvested tubular tissues, with a 10.23% increment in external diameter from 0 to 3.5 kg/cm^2^ pressurization.

In the dynamic group that received cyclic pressurization (pumping), a set including an inflatable silicone tube segment approximately 35 cm in length was used, and customized hand-made ports were placed on the two ends to connect to the pressurizing device (syringe pump) to drive the cyclic inflation of silicone tube. In the static group, a tube segment of approximately 7 cm was used. Silicone tube sets and segments were cleaned, sterilized with autoclave (120 °C, 20 min, 1.2 bar), and then dry preserved in an oven before autologous scaffold fabrication.

### Pressurizing device

V3 syringe pumps (Kloehn, U.S.) with a 2.5-mL or 5-mL syringe were used for reciprocate pumping processes to generate cyclic mechanical stimulation. The dynamic group were separated further into two groups with the maximum pressure set at 3.5 kg/cm^2^: the fast pumping group with frequency set at 30 cycle/min (0.5 Hz) and the slow pumping group with frequency set at 12 cycle/min (0.2 Hz). We monitored the pressure real-time using Labview custom software (National Instruments, U.S.) with a pressure monitor set, which includes a pressure transducer (Sensormate Enterprise Co., Taiwan) connected to a computer with A-D convert interface (USB-6210, National Instruments, U.S.). Tygon S-50-HL tubings (Saint-Gobain, France) were used for connecting the syringe pump and pressure transducer to the experiment animal. The connecting tubes were protected by light-weight covering coils to prevent damage from animal bites. Tubes were connected to each other with luer connectors.

### Animal subjects

The animal experiment protocol was reviewed and approved by the Institutional Animal Care and Use Committee of National Cheng Kung University (NCKU) and all methods were performed in accordance with relevant guidelines and regulations. We purchased male Sprague-Dawley rats aged 8–10 months (with body weights of about 700 g) from the NCKU Animal Center and gave them 13-hour-day-11-hour-night shifts in a 22 °C environment.

We anesthetized the rats with intraperitoneal injections of Zoletil 50 (25 mg tiletamine and zolazepam 25 mg/mL, Virbac, France) with the dosage of 100 µL per 100 g body weight. After anesthetization, we shaved the rat below its neck with an electric shaver before cleaning the area with a scrub brush and chlorhexidine gluconate 4% w/v cleaner twice, and then with a 7.5% povidone iodine cleaner. After that, the rat was dried, put on sterile sheet with an underlying heating pad to preserve body temperature, and then disinfected with iodine alcohol. A 5-mL sample venous blood was drawn from the external jugular vein through a 1-cm skin cut and preserved in an acid citrate dextrose-A solution (trisodium citrate, 22.0 g/L; citric acid, 8.0 g/L; and dextrose 24.5 g/L, 1.5 mL, BD, U.S.) in a blood collecting tube. Before drawing blood, we added antibiotics, 6 mg gentamicin sulfate (Panbiotic Laboratories, Taiwan) +40 mg cefazolin (Yung Shin Pharmaceutical Industrial Co., Taiwan) to the blood collecting tube to avoid bacterial growth. After blood collection and hemostasis, we closed the skin wound with 4–0 nylon suture (Unik, Taiwan).

### Whole blood scaffold fabrication

**Figure 1:**
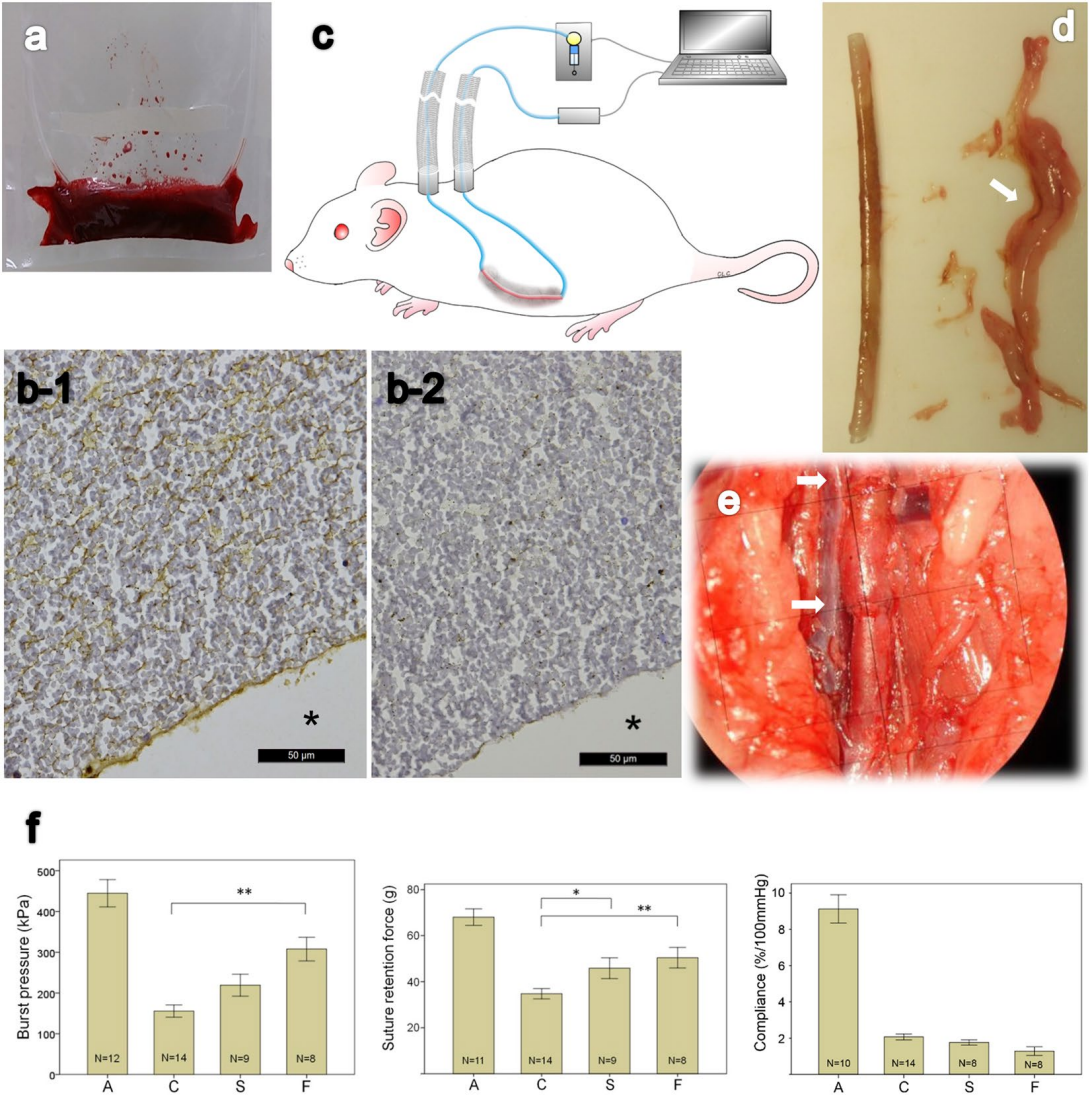
(**a**) Whole blood scaffold fabricated in a sterile container, the silicone tube extends from both sides of the scaffold. (**b-1**) IHC for LPS of the scaffold, brown color indicates LPS and* indicates luminal area where the silicone tube was removed. (**b-2**) Negative control of IHC with isotype non-immune IgG. Scale bar indicates 50 μm. (**c**) Animal experiment setting shows a syringe pump and a pressure transducer connected to both ends of the tubing system, with a PC utilized for manipulating and monitoring. Blue line inside the rat shows the subcutaneous silicone tube, and red line shows the part overlaid with the LPS-whole blood scaffold (displayed in gray) inside peritoneal cavity. (**d**) Tubular tissue forming on the implanted silicone tube (both cutting edges can be seen) after 24-day incubation in the peritoneal cavity; adhering mesentery tissues were separated at right side where supplying blood vessels showed by an arrow. (**e**) Tubular tissue implanted as interpositional aortic graft, arrows showing upper and lower anastomosis. (**f**) Results of mechanical tests of the tubular tissues. A: aorta, C: static group, S: slow-pumping group, F: fast pumping group; *p < 0.05, **p < 0.005; error bar: ±1 SE. There is no significance between experimental groups in the compliance test.

We added 10 μg of LPS from *Escherichia coli* 0111:B4 (L4391, Sigma-Aldrich Co., U.S.) into the blood collecting tubes, gently (low speed) vortex mixed them for 3 minutes and then placed them on a blood tube roller mixer until further use. The silicone tube set (including two ports, for the dynamic group) or tube segment (for the static group) to be implanted, was first treated in the plasma cleaner (PDC-32G, Harrick Plasma, U.S.) for surface modification in order to increase hydrophilicity, with oxygen pressure set at 0.2 to 0.3 mmHg (Torr) and flow rates below 10 mL/min, under the “High” power setting of the plasma machine for 7 minutes. The tube set or segment was then put on a sterile sheet lining the plasma cleaner chamber. After oxygen plasma treatment, we placed the dynamic group’s tube set, or the static group’s tube segment in a hanging sterile plastic container designed for molding the scaffold. After securing the position of the tube set or segment in the container, we added the LPS-antibiotics-blood mixture into the container with a sterile dropper. Then, a mixture of 0.87 mL 10% CaCl_2_ sterile water solution made with calcium chloride dihydrate (C3881, Sigma, U.S.) and bovine thrombin 150 u (112374, Merck, Germany or T4648, Sigma, U.S.) was evenly distributed in the container with a 1-mL-tip pipet across the whole fabrication area. The whole mixture was kept stationary for more than 15 minutes for the scaffold to “set” (Fig. 1a).

### Quantification of lipopolysaccharide in the whole blood scaffold

We measured the effective LPS amount in the fabricated LPS-whole blood scaffold using ToxinSensor chromogenic LAL endotoxin assay kit (GenScript, China). The sample of LPS-added and LPS-free whole blood scaffold to be analyzed was dried on a tissue paper to remove excessive exudate/fluid and then weighed. Samples were homogenized with a sonicator (Soniprep 150, MSE, UK) in 2 mL LPS-free water, then with further dilution. The final dilution ratio of the whole blood scaffold using LPS-free water for LAL test was 1:4000. Analysis was performed according to the operation manual of the kit. The absorbance was read using a microplate spectrophotometer (Epoch 2, Biotek, U.S.).

### Autologous re-implantation of the scaffold into the peritoneal cavity

In a supine position, rats were disinfected with iodine alcohol again. We made two longitudinal skin incisions approximately 3 cm in length on both sides in the abdominal area, distanced about 6 to 7 cm apart. We cut through underlying abdominal muscle layers until the peritoneal cavity was reached. Then, we took the tube set (or segment) with the surrounding whole blood scaffold out of the container and placed it into the peritoneal cavity through the two abdominal incisions, making sure not to damage the fragile and jelly-like scaffold or let the inner silicone tube wear out or fall off the scaffold. The position of the upper end of the scaffold was approximately 1 cm below the lowest boarder of the liver, with an intraperitoneal segment (with scaffold) of about 6 to 7 cm, not too deep as to interfere with peritoneal contents. For the static group, we put the scaffold with inner silicone tube through the two incisions and then anchored the two ends of the tube to the abdominal muscle layers with 4-0 nylon suture (Unik, Taiwan). The abdominal muscle layers were subsequently closed with 4-0 absorbable polyglycolic acid suture (VT174, Unik, Taiwan). For the dynamic groups, we closed both abdominal incisions with the same absorbable suture, leaving two extended segments of the tube set out of the peritoneal cavity bilaterally. Then two ends of the tube (with ports) were tunneled subcutaneously to the back, with the two ports fixed to each other subcutaneously with 3-0 silk suture (Unik, Taiwan), leaving two openings (ports) out of the skin on the back. After the operation, we put the rat on normal (prone) resuscitation position and gave a 7-day recovery period before subsequent pumping process.

### Connection of animals to pressurizing devices

We used a custom-made harness for fixing pipelines onto the rat and adjusted the tightness of the harness so that it would not distress the rat. One port on the back of the rat was used to connect to the syringe pump, and the other to connect to the pressure transducer (Fig. 1c). After the two ports were connected, we infused tap water into the tubing system with a syringe. Because the whole tubing system was water impermeable, the infusion of water posed no risk of infection. Following the evacuation of remnant air in the tubing, we applied the two light-weight metal protecting coils to the tubing system to prevent animal bites. We set the syringe pump to work cyclically with a maximum pressure of about 3.5 kg/cm^2^ in each cycle. This pumping process was applied for 8 hr/day for 16 consecutive days. During the process, the rats were able to ambulate and eat ad lib. After the daily pumping process, the rats were removed from two connecting pipelines and given an opportunity to rest. After the 16-day stimulation, the rats were given a 1-day rest before being euthanized for tissue samples or subjected to further autologous implantations.

### Tissue tube harvest

After incubating in the peritoneal cavity for 24 days (7 days post operation, 16 days pressurization or static, and 1 day of rest), the rats were euthanized by CO_2_. We then performed a midline laparotomy, cutting out the implanted silicone tube along with surrounding tubular tissue. With the silicone tube still inside, we removed redundant adherent mesenteric tissues using a surgical blade to prevent the possibility of interfering measurements of the outer diameter (OD) in the mechanical tests. When making short tissue segments for histology analyses, we kept the silicone tube for maintaining the tubular structure during histological fixation. We preserved the samples in 10% neutral buffered formalin solution overnight before histological processes. Infected tissue tubes, which showed formation of pus, apparent thickening, or incomplete or discrete tissue, were deemed to be failures. A segment of approximately 10 mm long was used for mechanical tests. The aorta of the same rat was also harvested for mechanical tests and analyses of ECM and proteins. Samples were stored at −80 °C before the protein/ECM analyses.

### Mechanical tests

We tested the mechanical properties of the tissue tubes using a custom made pressure-diameter mechanical tester set consisting of a motion control system (MID-7604 and PXI-7330, National Instruments, U.S.) with a stepper motor, a syringe pump (KDS-210, KD Scientific, U.S.), a pressure transducer (Model 209, 0–5 psig, Setra, Box-borough, U.S.), a force load cell (LTS-200GA, Kyowa, Japan), a 1394 charge-coupled device camera (656 × 492, Stingray F033B, Germany), a television lens (HF25HA-1B, Fujinon, Japan), and a custom built loading frame^22^. A 10-mm segment of the tissue tube or infrarenal abdominal aorta was cannulated with Luer adaptors using 3-0 silk suture (Unik, Taiwan) and coupled with the loading frame. The tissue tube was immersed in a transparent container filled with normal saline at room temperature. Before the test, we expelled the air in the tubing and then stretched the tissue tube to the initial measured axial force of about 10 g to keep the tissue tube straight and prevent it from buckling (which might hamper the OD measurement) during testing cycles. The axially constrained tissue tube was then subjected to cyclic pressurization between 10 and 160 mmHg for 10 cycles, with a syringe pump at a flow rate of 0.2 mL/min. After compliance testing cycles, the measured axial force was 3 to 5 g. We recorded the average data from the loading phase of last three cycles and calculated the compliance using the following formula:

$${\rm{Compliance}}\,( \% \,{\rm{per}}\,100\,\mathrm{mmHg})=({{\rm{D}}}_{{\rm{systolic}}}-{{\rm{D}}}_{{\rm{diastolic}}})\times {10}^{4}/{{\rm{D}}}_{{\rm{diastolic}}}({{\rm{P}}}_{{\rm{systolic}}}-{{\rm{P}}}_{{\rm{diastolic}}}),$$

where P_systolic_ and D_systolic_ are systolic pressure and the corresponding OD of the tissue tube, and P_diastolic_ and D_diastolic_ are the diastolic pressure and the corresponding OD of the tissue tube. We calculated the compliance using 140 mmHg as the systolic pressure and 70 mmHg as the diastolic pressure.

The burst pressure and suture retention force were tested using the same custom-built mechanical tester, except that a 100-psi pressure transducer (Model 80 A, 0–100 psi, Sensormate, Taiwan) was used for a larger maximum pressure. The burst pressure was the measured maximum pressure by pumping at a flow rate of 0.2 mL/min before breaking the tube. The suture retention force was tested on a 5-mm tissue tube or abdominal infrarenal aorta, with one end fixed on the tester. With a tapper point needled 6-0 polypropylene suture (Unik, Taiwan), we made a knot 1 mm from the edge of the tissue tube and fixed it to a pre-made loop (made from 3-0 silk suture) that was fixed to the force load cell. The set was stretched at an extension rate of 15 mm/min until the suture broke through, and we recorded the maximum force measured before the breakage. Two tests were performed for each tissue tube, and the mean was recorded. After mechanical tests, we stored the tissues at −80 °C.

### Western blot

Samples were thoroughly homogenized with a radioimmunoprecipitation assay buffer and protease inhibitor (Complete Ultra, Roche, Switzerland) and then centrifuged (4 °C, 14000 rpm) to acquire suspension for analysis. We quantified the protein contents using the DC^TM^ protein assay kit (Biorad, U.S.). Samples were then heated for 5 min at 95 °C, and equal amounts of protein (40 µg for each load) were electrophoresed on a SDS-PAGE gel and then transferred to polyvinylidene difluoride membranes. Conjugated 1st antibodies were as follow: mouse monoclonal alpha-smooth muscle actin (1:100) (Abcam, ab7817), mouse monoclonal anti-smooth muscle myosin heavy chain 11 antibody (1:125) (Abcam, ab683), rabbit monoclonal calponin-1 antibody [EP798Y] (1:10000) (Genetex, GTX61419), and rabbit anti-smooth muscle myosin IgG (1:75) (Biomedical Technologies Inc., BT-562). The 2nd antibodies were goat polyclonal anti-rabbit IgG (HRP) (Abcam, ab97051), Genetex polyclonal goat anti-rabbit IgG antibody (HRP) (Genetex, GTX213110-01), and Genetex goat polyclonal anti-mouse IgG (HRP) (Genetex, GTX213111-01). Beta-actin was chosen as loading controls with mouse monoclonal anti-beta actin antibody (Abcam, ab8224, 1:8000). We used the WesternBright ECL^TM^ western blot detection kit (Advansta, U.S.) and recorded chemiluminescent signals using BioSpectrum AC imaging system (UVP, U.S.). Densitometric analyses were performed using Image J software (NIH, U.S.), and the value of each band of specific antibody was normalized to the corresponding value of beta-actin.

### Elastin and collagen quantification

We quantified elastin and collagen using the Sircol and Fastin kits (Biocolor, U.K.). For elastin analysis, tissue to be analyzed was rinsed with phosphate-buffered saline for three times for cleaning, stored at −80 °C, and then lyophilized. We extracted 2 mg dry weight of the tissue under heating at 100 °C with 0.25 M oxalic acid for 120 min and analyzed the obtained suspension according to the operation manual. Two extractions were performed for each specimen, and the results were summed up. The third extraction was performed once and showed minimal elastin content (data not shown), so we choose the two-extraction procedure. For collagen analysis, samples were rinsed with phosphate-buffered saline for three times and lyophilized as previously described, and then 2 mg dry weight of the tissue was extracted and placed in 0.5 mg/mL pepsin and 0.5 M acetic acid for 30 min before thorough homogenization and standard processes described in the manual. We expressed the data as µg elastin or collagen per mg dry weight.

### Autologous aortic interpositional re-implantation

After the dynamic or static peritoneal incubation period, we anesthetized the rat with intraperitoneal injections of Zoletil 50 (25 mg tiletamine and zolazepam 25 mg/mL, Virbac, France) with a dosage of 100 μL/100 g body weight. The rat was then put on a heating pad to avoid hypothermia. After aseptic pre-operation procedures, we performed a midline laparotomy to expose the implanted silicone tube and its surrounding tissues in the peritoneal cavity. Adhesion of mesentery tissues were released with bipolar electrocauterization for hemostasis. We cut the intraperitoneal segment of silicone tube along with the outer tissue tube from both sides of the abdominal wall. The inner silicone tube was discarded, and a segment of the outer surrounding tissue tube about 8 mm in length was trimmed and kept in a heparinized normal saline (100 U/mL saline) before implantation. The infrarenal aorta was released from the neighboring inferior vena cava. We then clamped the free infrarenal aorta on both the proximal and distal parts with a double-clamp, cut both ends, and flushed the two cut ends with heparinized saline. The harvested tissue tube was sewn first on the proximal end, with 10 to 11 simple stitches of 10-0 nylon suture (Unik, Taiwan), and then the distal end. After anastomosis of both ends of the tissue tube, the distal clamp was removed first, and then the proximal clamp. After securing hemostasis, we flushed the peritoneal cavity with 10 mL pre-warmed saline twice. Abdominal muscle layers were closed by 4-0 absorbable polyglycolic acid suture (Unik, Taiwan), and skin was closed with 4-0 nylon (Unik, Taiwan). For the dynamic group, subcutaneous silicone tube and ports on the back were removed, and the wound on the back was closed with 4-0 nylon suture. After the operation, the rat was put under a heating lamp before awakening, and no anti-platelet or anti-coagulant was administered.

### Ultrasonographic imaging analysis of the implanted vascular grafts

We confirmed the patency of the graft using ultrasonography at 1 week and 1, 2, and 4 months after the graft surgery. As previously described, the rats were subject to anaesthetization and midline laparotomies. The grafted tissue tube and adjoining upper and lower infrarenal aorta were thus exposed. We then used high-frequency micro-imaging ultrasonography model Vevo 770 with the scanhead model RMV 704 or RMV 708 (VisualSonics, Canada) for measurements and applied power Doppler mode to check the patency of the grafted tissue tube. Pictures and video footages were recorded, and video footage was analyzed frame by frame with screen pixel measurements to acquire the minimum and maximum internal diameter (ID) at the midpoint of implanted tissue tube and adjoining proximal aorta.

The indicators include the following:

$$\begin{array}{c}\mathrm{implant}-\mathrm{aorta}\,{\rm{compliance}}\,{\rm{ratio}}=\\ \,\frac{({\rm{maximum}}\,{\rm{implant}}\,{\rm{ID}}-{\rm{minimum}}\,{\rm{implant}}\,{\rm{ID}})/{\rm{minimum}}\,{\rm{implant}}\,{\rm{ID}}/{\rm{\Delta }}\mathrm{pressure}}{({\rm{maximum}}\,{\rm{arota}}\,{\rm{ID}}-{\rm{minimum}}\,{\rm{arota}}\,{\rm{ID}})/{\rm{minimum}}\,{\rm{arota}}\,{\rm{ID}}/{\rm{\Delta }}\mathrm{pressure}}\end{array}$$

Because all the IDs had the same unit and Δpressure is the same in numerator and denominator, the units are cancelled out during the calculation, and so the implant-aorta compliance ratio has no unit.

$${\rm{implant}}-{\rm{aorta}}\,{\rm{minimum}}\,{\rm{ID}}\,{\rm{ratio}}={\rm{minimum}}\,{\rm{implant}}\,{\rm{ID}}/{\rm{minimum}}\,{\rm{aorta}}\,\mathrm{ID},$$

$${\rm{implant}}-{\rm{aorta}}\,{\rm{maximum}}\,{\rm{ID}}\,{\rm{ratio}}={\rm{maximum}}\,{\rm{implant}}\,{\rm{ID}}/{\rm{maximum}}\,{\rm{aorta}}\,{\rm{ID}}{\rm{.}}$$

The two indicators above were used to evaluate dilation of the implant as compared to its proximal adjoining aorta at the diastolic (minimum ID ratio) and systolic (maximum ID ratio) status, respectively.

After the measurements, we euthanized the rat and removed the tissue tube with its adjoining proximal and distal aorta for further histological analysis.

### Histology

Samples were fixed in a 10% neutral buffered formalin solution overnight, dehydrated through a series of graded alcohol, and then embedded in paraffin. We made 5-µm sections for histology analysis and stained them with hematoxylin-eosin stain, Masson trichrome stain, and Verhoeff-van Gieson elastin stain. For all immunohistochemical (IHC) staining, except for LPS, negative control and CD68 staining, the sections were subjected to microwave treatment in a 10 mM (pH 6.0) citrate buffer for antigen retrieval. We incubated sections with the following primary antibodies: mouse monoclonal anti-alpha smooth muscle actin (aSMA, Abcam ab7817, dilution rate 1:8000), rabbit monoclonal anti-calponin-1 (Genetex GTX61419, dilution rate 1:8000), mouse monoclonal anti-smooth muscle myosin heavy chain 11 antibody (Abcam ab683, dilution rate 1:16000), and rabbit polyclonal anti-Von Willebrand factor antibody (VWF, Abcam ab6994, dilution rate 1:3200) for 1 hour at room temperature. Then the sections were incubated with biotinylated anti-mouse or anti-rabbit secondary antibody for 30 minutes, followed by streptavidin conjugated horseradish-peroxidase complex for 30 minutes. The sections were then incubated with diaminobenzidine chromogen for 3 to 5 minutes. For IHC staining of LPS, negative control of LPS and CD68, mouse monoclonal anti- E. coli LPS (ab35654, 1:100 dilution, Abcam), mouse monoclonal IgG2b kappa isotype control antibody clone MPC-11 (1:100 dilution, Stemcell, Canada), mouse monoclonal anti-CD68 antibody [KP1] (ab955,dilution 1:100, Abcam) was used correspondingly as the primary antibody. The procedures were done with the Bond-Max Automated IHC stainer (Leica Biosystems Newcastle Ltd., Australia). Tissues were deparaffinized with xylene and pre-treated with the Epitope Retrieval Solution 2 (EDTA, pH 9.0) at 100 °C for 30 min, followed by primary antibody incubation at room temperature for 30 min. To enhance polymer binding, we incubated with post primary (Leica Biosystems Newcastle Ltd., U.K.) at room temperature for 8 minutes. The anti-mouse/rabbit poly-HRP secondary antibody was incubated at room temperature (Leica Biosystems Newcastle Ltd., U.K.) for 8 min. Hydroperoxide blocking was incubated for 5 min using the Bond Polymer Refine Detection Kit (Leica Biosystems Newcastle Ltd., U.K.) and developed with diaminobenzidine chromogen. Counterstaining was carried out with hematoxylin.

We obtained histological images using an optical microscopy (DM2500P, Leica, Germany or Axio Scope.A1, Zeiss, Germany) with a CCD camera (DFC295 digital camera, Leica, Germany) or a DSLR camera (Canon 77D, Japan).

The thickness of the wall of harvested tubes in the second operation was calculated using at least 4 measurements for each tube, and the thickest part of the tube was excluded.

### Image analysis of cellular morphology

We used ImageJ software (NIH, U.S.) to analyze the aspect ratio (AR) of red-pink colored staining under Masson’s trichrome stain which comprises cytoplasm in the tissue wall after 24 days of intraperitoneal culture. Images to be analyzed were cross-sections of tissue tubes, acquired with 20X objective lens, first cropped to exclude the part outside the wall structure, than color thresholded using HSB (hue, saturation, brightness) mode, with red-pink color component to be cropped by adjusting individual HSB threshold. Then we used “analyze particles” process with setting of size of 0-infinity, circularity of 0–1. Each sample of static and fast pumping groups was analyzed for at least 3 times from 3 different sites of the sample, and the average was calculated.

### Statistical analysis

We performed statistical analyses and plotting using IBM SPSS statistics software Version 20 (IBM, U.S.). Data were first evaluated by the Shapiro-Wilk normality test. For comparisons among values with p > 0.05 for normality tests, ANOVA with least significant difference post hoc comparisons were performed. For those with at least one group with p < 0.05 for the normality test, the Kruskal-Wallis test with pair-wise comparison was performed. Data are presented as mean ± SE (standard error).

## Results

### Whole blood scaffold

**Figure 2:**
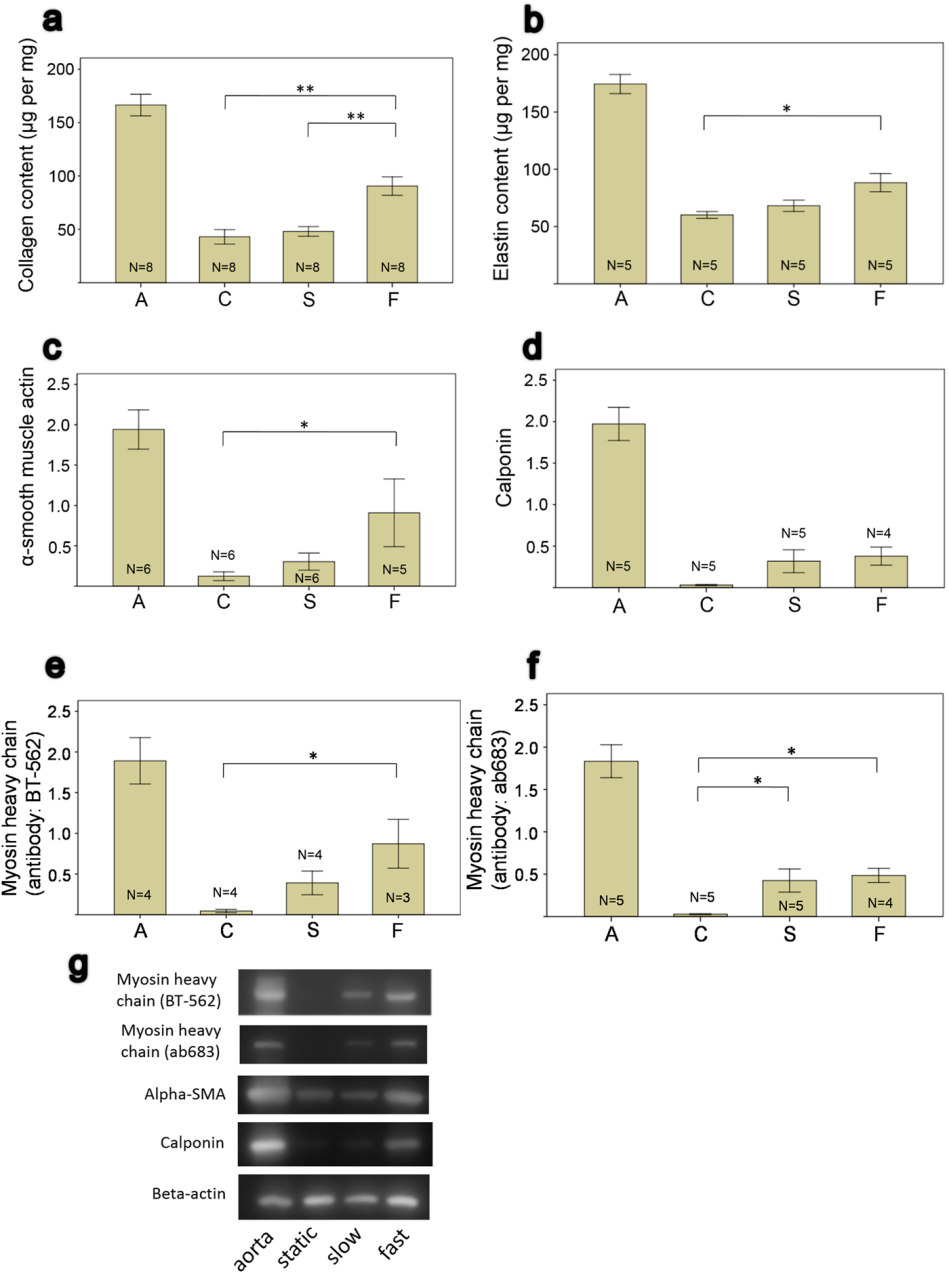
Compositional analysis of tissue tubes incubated in the peritoneal cavity after 24 days. (**a,b**) ECM analysis, data showing μg per mg of dry-weighted samples. (**c–f**) Western blot analysis. A = aorta, C = static (no pumping), S = slow-pumping, F = fast pumping. Significance between experimental groups are denoted as *P < 0.05, **P < 0.005. Error bar: ±1 SE. (**g**) Cropped images of western blot. Full-length blots/gels are presented in Supplementary Information.

IHC staining of the LPS of the blood scaffold showed that the applied LPS was distributed throughout the scaffold and luminal surface (Fig. 1b-1), negative control with non-immune isotype IgG was shown as Fig. 1b-2. The measured LPS level using LAL test in the LPS-added whole blood scaffold was 3155 ± 191 EU(endotoxin unit)/ml (n = 3); the non-LPS added whole blood scaffold was 200 EU/ml (n = 1).

### Tissue tube harvest

After 24 days of incubation inside the peritoneal cavity, the whole blood-LPS mixture scaffold turned into a thin semi-transparent-brownish colored tissue tube, covered with mesentery tissue (Fig. 1d). Whereas some mesentery tissue adhered to the tissue tube, little intestinal adhesion was noticed. The tissue tube could be easily detached from the inner silicone tube, but it was soft and flaccid without the ability to uphold its tubular shape. The overall yield rate of tissue tube (excluding infection) was 81.1% with the LPS-whole blood scaffold.

The thickness of the wall was 114.19 ± 3.00 µm (n = 3) in the aorta, 157.27 ± 11.53 μm (n = 9) in the harvested tissue tube in the static group, 99.58 ± 12.15 μm (n = 7) in the tubes in the slow pumping group, and 93.62 ± 13.97 μm (n = 5) in the fast pumping group. The differences between the static group and the slow pumping group (p = 0.002) and fast pumping group (p = 0.002) reached statistical significance.

### Mechanical tests

The tissue tubes incubated *in vivo* for 24 days showed an increasing trend in the burst pressure from the static group (155.62 ± 15.02 kPa, n = 14), the slow pumping group (219.1 ± 26.89 kPa, n = 9), the fast pumping group (307.68 ± 29.1 kPa, n = 8), to the native aorta (444.7 ± 33.46 kPa, n = 12) (Fig. 1f). Likewise, the measured suture retention force was 34.75 ± 2.2 g (n = 14) in the static group, 45.83 ± 4.51 g (n = 9) in the slow pumping group, 50.41 ± 4.45 g (n = 8) in the fast pumping group, and 68.02 ± 3.61 g (n = 11) in the native aorta. However, the compliance in the native aorta was 9.13 ± 0.78%/100 mmHg (n = 10), while it was 2.06 ± 0.16%/100 mmHg (n = 14) in the static group, 1.76 ± 0.14%/100 mmHg (n = 8) in the slow pumping group, and 1.28 ± 0.24%/100 mmHg (n = 8) in the fast pumping group.

### Elastin and collagen content

There was an increasing trend in collagen contents from the static group (42.96 ± 6.74 μg/mg, n = 8), to the slow pumping group (48.02 ± 4.56 μg/mg, n = 8), the fast pumping group (90.5 ± 8.63 μg/mg, n = 8), and the native aorta (166.44 ± 10.11 μg/mg, n = 8) (Fig. 2a). Likewise, the elastin content was 60.11 ± 2.94 μg/mg (n = 5) in the static group, 68.14 ± 5.01 μg/mg (n = 5) in the slow pumping group, 88.27 ± 7.92 μg/mg (n = 5) in the fast pumping group, and 174.44 ± 8.35 μg/mg (n = 5) in the native aorta (Fig. 2b).

### Western blot

Compared to the static group, the fast pumping group had increased initial smooth muscle differentiation markers—aSMA (Fig. 2c). The pumping groups also had increased calponin, but the differences did not reach statistical significance (Fig. 2d). For the higher smooth muscle differentiation marker, smooth muscle myosin heavy chain (Fig. 2e,f), and two antibodies (ab683 and BT-562) were significantly higher in the fast pumping group when compared to the static group, but only ab683 was significantly higher in the slow pumping group than the static group. Representative images of western blot are shown in Fig. 2g.

When the densitometric values of Western blot were normalized to beta-actin, aSMA was 1.94 ± 0.24 (n = 6) in the native aorta, 0.12 ± 0.05 (n = 6) in the static group, 0.3 ± 0.11 (n = 6) in the slow pumping group, and 0.91 ± 0.42 (n = 5) in the fast pumping group. Calponin was 1.97 ± 0.2 (n = 5) in the native aorta, 0.03 ± 0.01 (n = 5) in the static group, 0.32 ± 0.14 (n = 5) in the slow pumping group, and 0.38 ± 0.11 (n = 4) in the fast pumping group. Myosin heavy chain (antibody of BT-562) was 1.89 ± 0.29 (n = 4) in the native aorta, 0.04 ± 0.02 (n = 4) in the static group, 0.39 ± 0.15 (n = 4) in the slow pumping group, and 0.87 ± 0.3 (n = 3) in the fast pumping group. Myosin heavy chain (antibody of ab683) was 1.83 ± 0.19 (n = 5) in the native aorta, 0.03 ± 0.01 (n = 5) in the static group, 0.42 ± 0.14 (n = 5) in the slow pumping group, and 0.48 ± 0.08 (n = 4) in the fast pumping group.

### Autologous aortic interpositional re-implantation

About one-third of the rats survived the autologous aortic grafting surgery. These rats were not subject to additional treatment to the luminal surface of tissue tube, nor the application of antiplatelet/anticoagulant after the operation, except the short period for the tissue tube dipping in the heparinized normal saline before grafting. Once they survived this critical period, most of rats lived through the experiment. A total of 24 rats completed the grafting experiment and survived for the following scheduled periods: 1 week (N = 2; 1 in the fast pumping group, 1 in the static group), 1 month (N = 1, in the static group), 2 months (N = 1, in the static group), 4 months (N = 20; 5 in the static group, 8 in the slow pumping group, 7 in the fast pumping group).

### Image analysis of cellular morphology

Under Masson’s trichrome staining, the AR of cytoplasm of the tissue tube after 24 days of intraperitoneal culture was smaller in the static group (n = 6) than in the fast pumping group (n = 5) (2.05 ± 0.07 vs. 2.45 ± 0.18, p < 0.05 for the Mann-Whitney U test).

### Histology

**Figure 3:**
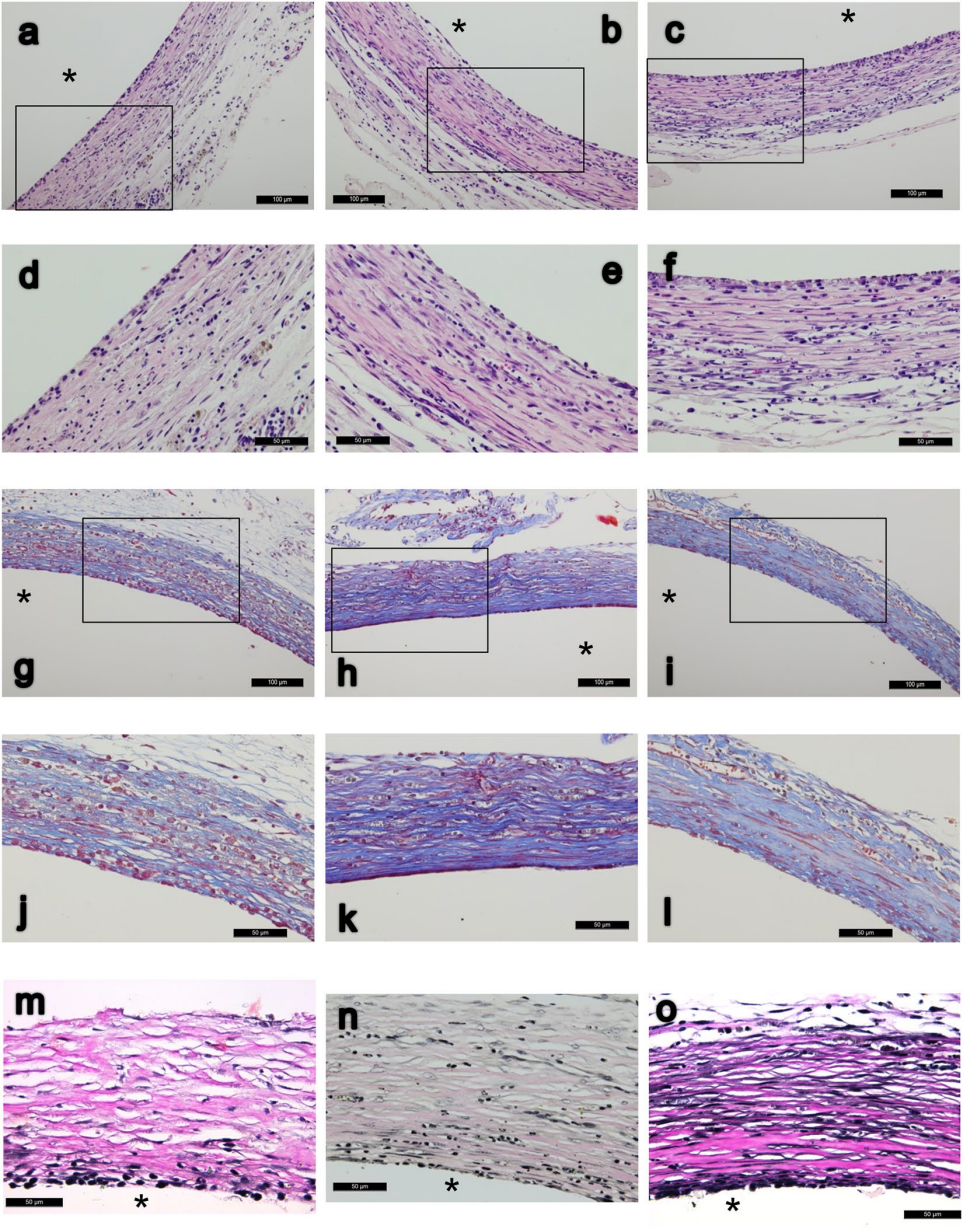
(**a–f**) H&E stain of tissue tubes forming on implanted silicone tube of static (**a,d**), slow-pumping (**b,e**) and fast-pumping (**c,f)** after 24 days of incubation in the peritoneal cavity; (**d–f**) are close-ups of (**a–c**) accordingly. (**g–l**) Masson’s trichrome stain of static (**g,j**), slow-pumping (**h,k**) or fast-pumping group (**i,l**) which (**j–l**) are close-ups of (**g–i**) accordingly. (**m–o**) Verhoeff–Van Gieson elastin stain of static (**m**), slow-pumping (**n**) and fast-pumping (**o**). *Indicates the removed silicone tube; scale bar as indicated. All cross-sections.

**Figure 4:**
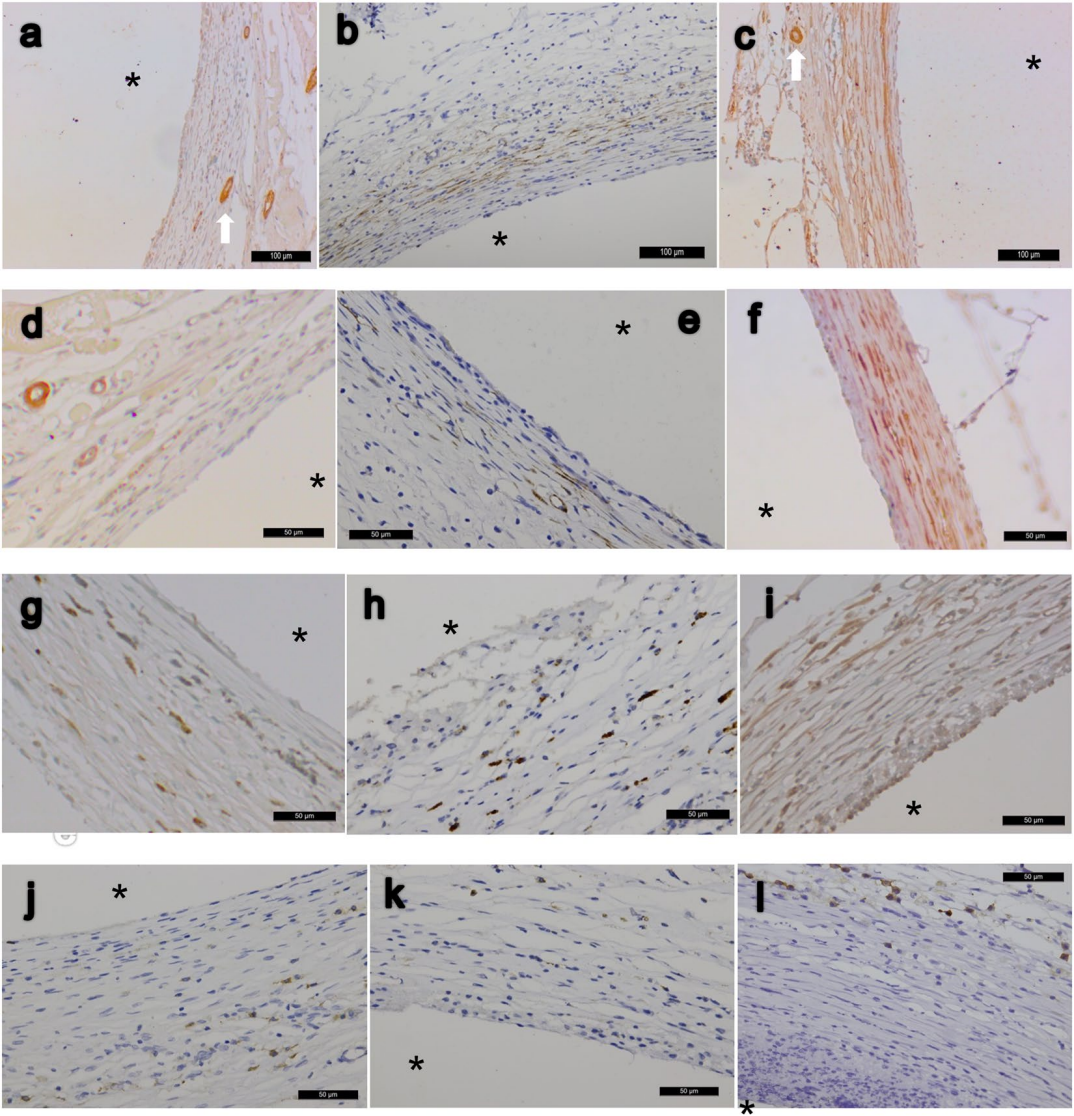
IHC stain of tubular tissues forming on implanted silicone tube after incubation of 24 days in the peritoneal cavity. (**a–c**) α-smooth muscle actin, static group (**a**), slow-pumping (**b**) and fast-pumping (**c**); capillary vessels (arrows) seen as positive control. There are apparent positive streaks/bundles of ECM staining (brown color) throughout the whole tubular wall in the pumping groups. (**d–f**) Calponin of static (**d**), slow-pumping (**e**) and fast pumping group (**f**). (**g–i**) Myosin heavy chain of static (**g**), slow-pumping (**h**) and fast pumping (**i**), with more positive staining spindle-like cells (brown color) in the pumping group. (**j**–**l**) CD68 of static (**j)**, slow-pumping (**k**) and fast-pumping (**l**) (brown color). Asterisks indicate luminal sides which the silicone tube is removed. Scale bars as indicated; all cross-sections.

Microscopically, the H&E staining were shown in Fig. 3a–f. Masson’s trichrome staining revealed more prominently elongated red-pinked cytoplasms in the pumping groups as the results of image quantification (Fig. 3g–l). Verhoeff-van Gieson elastin stain revealed more elastic fibers in the pumping groups as the results of elastin quantification (Fig. 3m–o). IHC stain of smooth muscle differentiation markers (aSMA, calponin, and smooth muscle myosin heavy chain) all revealed more abundant positive bundle/streak ECM pattern in the pumping groups (Fig. 4a–i). CD68, a marker of macrophage/monocyte, was found in the outer area of tissue tube (Fig. 4j–l). Remnant LPS was not apparent with the IHC staining (data not shown).

**Figure 5:**
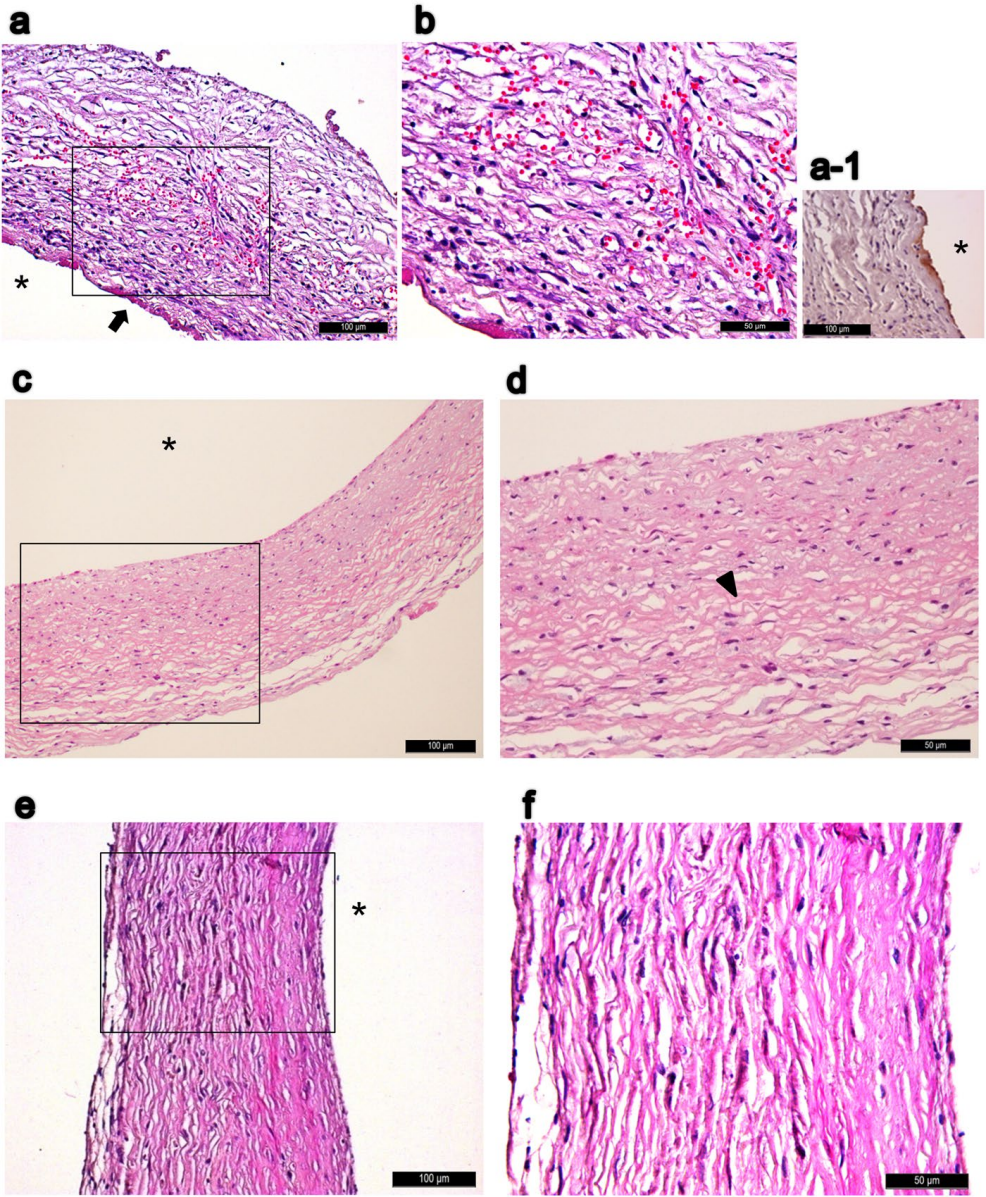
Remodeling process demonstrated by H&E staining of harvested static (no pumping) tissue tubes as infrarenal aorta interpositional implantation, after 1 week (**a,b,a-1**), 1 month (**c,d**) and 2 months (**e,f**). There was still inflammatory reaction after 1 week of implantation (**a,b**) which composed of spindle-like myofibroblasts, leukocytes and RBCs; partial thrombus can still be seen on the surface of lumen (arrow, **a**). Positive IHC staining of VWF (brown) at luminal surface after 1 week of implantation (**a-1**). (**c,d**) One month after the implantation, (**d**) is the close-up of (**c**); wave-like collagen component is shown (arrowhead, **d**), and no more noticeable remnant luminal thrombus. (**e,f**) Two months after the implantation; (**f**) is the close-up of (**e**). Asterisks indicate luminal side and scale bars as indicated. All cross-sections.

**Figure 6:**
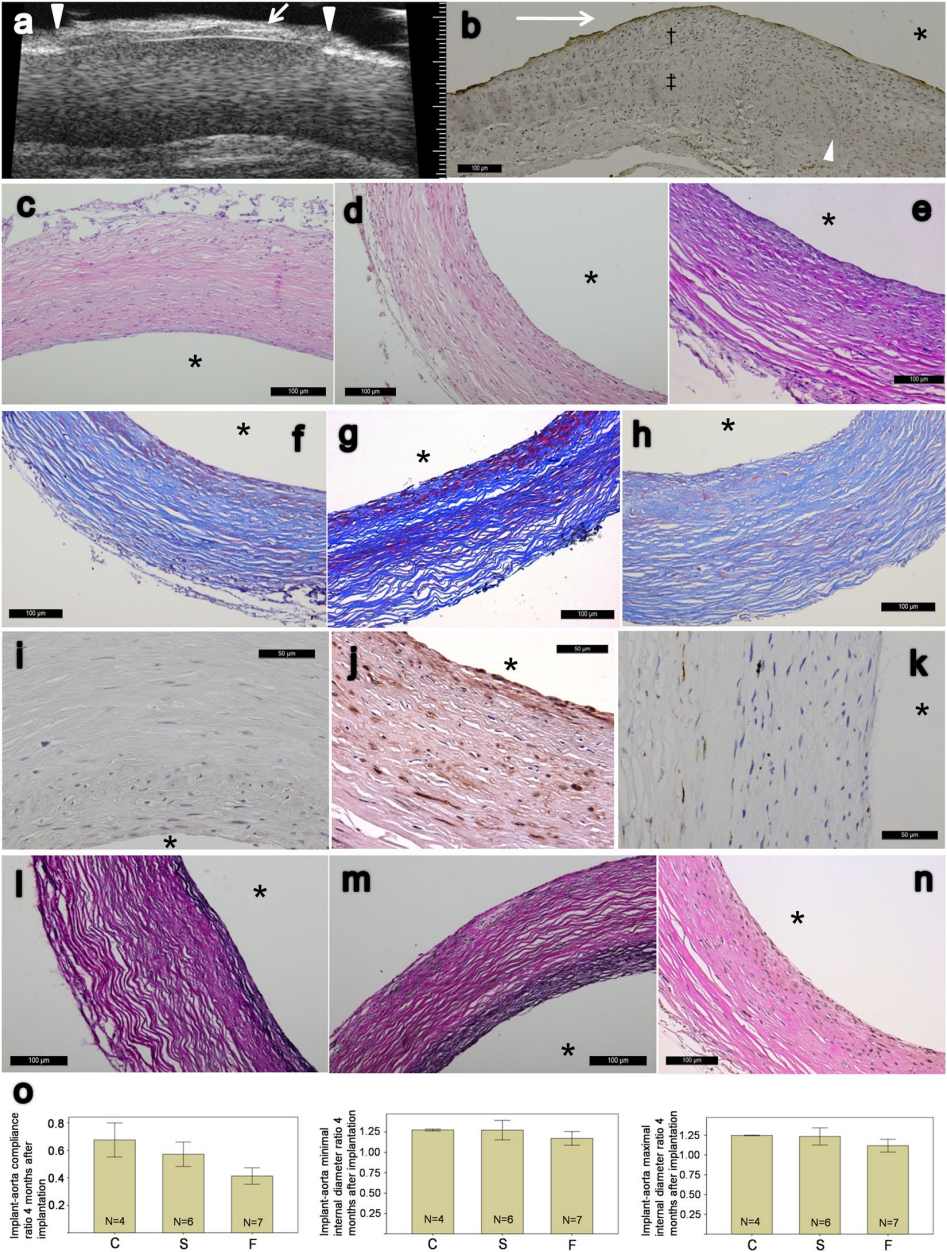
(**a**) Ultrasonography of implanted tubular tissue as an aortic graft after 4 months, arrowheads indicate anastomosis. Layer-like structure depicted as horizontal lines (arrow). Right major scale denotes 1 mm. (**b**) IHC stain of VWF, longitudinal section of the junction across aorta and implanted tissue tube after 4 months of implantation; blood flow comes from left to right indicated as arrow. †Denotes the junctional tissues in connection with the graft that covered and eventually merged with neighboring aorta, ‡Denotes native aorta and arrowhead denotes end of aorta. (**c–e**) H&E stain of 4 months after implantation of the static (**c**), slow (**d**) and fast pumping group (**e**). (**f–h**) Masson’s trichrome stain of 4 months after implantation of the static (**f**), slow (**g**) and fast pumping group (**h**). (**i–k**) IHC stain of myosin heavy chain after 4 months of implantation, static (**i**), slow (**j**) and fast-pumping group (**k**). **(l–n**) VVG elastin stain after 4 months of implantation, static (**l**), slow (**m**) and fast-pumping group (**n**). (**o**) Ultrasonographic analysis after 4 months post implantation, statistically insignificant between all groups of 3 kinds of measurements. Error bars denote ±1 SE. C = static, S = slow pumping, F = fast pumping. Stains are all cross-sections except (**b**); scale bars as indicated and asterisks denote luminal sides.

After one week of histological remodeling after being implanted as an interpositional aorta graft, there were still inflammatory reactions in the implant, which was composed of spindle-like myofibroblasts, leukocytes, and red blood cells (Fig. 5a,b). Thrombus could still be seen on the surface of lumen (Fig. 5a, arrow), and positive IHC staining of VWF could be seen on the luminal surface (brown, Fig. 5a–1). After one month of implantation, the cellularity became more homogenous without visible thrombi in lumen (Fig. 5c,d), and wave-like collagen fibers could be seen (arrowhead, Fig. 5d). After 2 months (Fig. 5e,f), the picture was closer to the 4-month implant. Grossly, unlike freshly tissue tubes that were harvested right after 24 days of peritoneal incubation, the tissue tubes that had been implanted as vascular grafts for four months showed gross elasticity and resiliently maintained the tubular shape against manual deformation upon their harvest. The ultrasonographic image before harvest was shown in Fig. 6a. After four months of implantation, positive IHC stains of VWF over luminal surface were shown in Fig. 6b. The junctional area across the neighboring aorta and the graft was covered with some vascular tissue (Fig. 6b, “†” mark) which was in connection to the graft and gradually merged with the aorta (Fig. 6b, “‡” mark); however the specific cellular origin of the junctional tissue and possible effects onto the graft remains to be investigated. H&E stains were shown in Fig. 6c–e. Masson’s trichrome stain revealed a wave-like collagen-dominant structure in the vascular graft (Fig. 6f–h), with a picture very different from that before implantation (Fig. 3g–l). However, the area of dense cellularity and area of loose cellularity (with spaces) could be clearly identified (Fig. 6g) and thus might cause the “layer-like” structure under ultrasonography (Fig. 6a, arrow). IHC stains of myosin heavy chain were shown in Fig. 6i–k, where positive stains of the fast pumping group (Fig. 6k) did not seem to exceed the slow pumping group (Fig. 6j). The same condition was observed for the VVG stains (Fig. 6l–n): there was only relatively sparse and thin elastic staining in the fast pumping group (Fig. 6n) as compared to the slow pumping group, in which elastic fibers scattered over the whole implant wall especially in the area close to the lumen (Fig. 6m). However these findings were still different from the typical elastic fiber expression in native large arteries.

### Ultrasonographic imaging and analysis of the implanted vascular grafts

Under ultrasonographic imaging, we inspected the patency of grafted tissue tubes with the power Doppler mode and observed full patency for all of them (Fig. 6a). No aneurysm formation or thrombus was identified in the 4-month grafts. Horizontal lines in the wall of implanted tissue tube were observed in the longitudinal view of ultrasonography (Fig. 6a, arrow). This might be correlated to the different density of the wall composition depicted in histology. Frame-by-frame sonographic image analysis of the 4-month grafts did not show any statistically significant differences among the groups in the three kinds of measurements (Fig. 6o). The graft-aorta compliance ratio after 4 months was 0.68 ± 0.12 (n = 4) in the static group, 0.57 ± 0.09 (n = 6) in the slow pumping group, and 0.41 ± 0.06 (n = 7) in the fast pumping group. The implant-aorta minimum ID ratio was 1.28 ± 0.01 (n = 4) in the static group, 1.27 ± 0.12 (n = 6) in the slow pumping group, and 1.17 ± 0.08 (n = 7) in the fast pumping group. The implant-aorta maximum ID ratio was 1.25 ± 0.00 (n = 4) in the static group, 1.23 ± 0.11 (n = 6) in the slow pumping group, and 1.12 ± 0.08 (n = 7) in the fast pumping group.

## Discussion

### Lipopolysaccharide

The overall yield rate of tissue tube was 81.1%, which was higher than those reported^16–18^.

In our preliminary study, we tried to add whole blood scaffolds (with antibiotics) over bare sterilized silicone tubes, apply oxygen plasma treatment to the tubes, and perform cyclic mechanical stimulation after the tubes were implanted in the peritoneal cavity. However, each of these processes alone or in combination could not stably yield tubular tissue surrounding the implanted silicone tube in the peritoneal cavity. In humans, for peritoneal continuous ambulatory peritoneal dialysis (CAPD), the silicone catheters should be implanted for at least 2 weeks before their first use^23^. If the formation of encapsulating tissue is inevitable, CAPD cannot be performed because the device will have outflow obstruction. LPS can increase the adhesion stimulated macrophages^24,25^ and result in morphologic changes such as increasing their roughness and diameter^25^, F-actin redistribution^25^, increasing actin polymerization^26^, and increasing cell spreading^25,26^ and filapodial projection^26^. We believed these changes play an important role in stabilizing the tissue surrounding the implanted silicone tube. Figure 4j–l shows the positive stain of CD68, which is a macrophage/monocyte marker and should be negative in normal mesothelium^27^, in the outer area of a tissue tube; this might explain the cell origin of the yielded tissue tube. It was reported that seeded human bone marrow mononuclear cells (BMCs) into the biodegradable scaffold secreted monocyte chemoattractant protein-1 (MCP-1), recruiting early host (SCID/bg mouse) monocytes and subsequently the scaffold transformed into functional blood vessels^28^. Hence the initial macrophages/monocytes recruitment using LPS might play a key role in our model, but other agents to recruit initial macrophages/monocytes such as modified synthetic TLR4 ligands could be considered in further studies. Due to concerns that inflammatory cytokines may be released from autologous blood cells during the manipulation of the whole blood or the operation, we did not use other pro-inflammatory cytokines.

In our preliminary study, we tried several dosages of LPS, ranging from 2 μg to 15 μg, and the 10 μg dosage had the best effect, while lower dosage resulted in thin and incomplete yield of tubular tissue and higher dosage resulted in over-thickness that was inadequate for transplantation. LAL test showed the active LPS was about 15.8% of added LPS in the whole blood scaffold (assume 10EU/ml = 1.0 ng/ml, we added 10 µg in 5 ml blood = 20000EU/ml, 3155/20000 = 15.8%), and since many agents or containers used in the experimental process was not LPS-free, we detected an LPS level of 200EU/ml in the non-LPS added blood scaffold. There are natural endotoxin inactivation mechanisms *in vivo*, including plasma protein^29^ which may explain the decreased LPS measurement in the blood scaffold and should be considered when adjusting the dosage of LPS in future *in vivo* studies. In addition, during the surgical implantation of the LPS-blood scaffold, the fluid or exudate over the scaffold could contain free, “unneutralized” LPS which might cause adverse effect and should be a concern. A previous study found that LPS stimulation could significantly enhance secretion of uPA, EGF-VEGF, FGF-2, FGF-4, and angiopoietin-2 in cultured skin-derived stem cell, and thus VEGF, thromborespondin-2, TGF-beta increased, and increased vascularization was also found^30^. Another study used a co-culture system consisting of primary human osteoblasts and outgrowth endothelial cells and found that LPS enhanced microvessel formation, with increased levels of ICAM-1, E-selectin, IL-6, IL-8, VEGF, and PDGF-BB^31^. In addition, the expression of TLR4 in adult stem and progenitor cells^32^ might suggest the potential use of LPS in the field of regenerative medicine.

### Whole blood scaffold

In our study, the thickness of wall of the tissue tubes had some variation, this might be caused by the irregular geometries of the whole blood scaffold, or the effect of uneven contacts with peritoneal contents when the rat stood up or moved. The appendant mesentery tissue (Fig. 1d) on the tissue tube made it difficult to invert the outer part inside to serve as the luminal part because lumen might become irregular. In our study, failures to yield tissue tube might be due to the implanted LPS-whole blood scaffolds dislodging from the silicone tube, owing to their soft and fragile nature. This should be addressed in future studies.

Fibrin scaffolds have been used to produce TEBVs^14,33–35^ or tissue-engineered heart valves^15^ *in vitro*. Ideally, fibrin scaffolds need to be degraded completely and replaced by collagen secreted by cultured cells before they have sufficient mechanical properties. Fibrin remnants still exist in fibrin-based TEBV even after a long duration of culture^14,33,35,36^, and complete degradation of the fibrin scaffold before sufficient ECM being secreted by cultured cells, which comprise the main structure of the TEBV, will result in failure of the graft. We did not add a regimen to interfere with fibrinolysis in the whole blood scaffold, because the peritoneal cavity carries the property of fibrinolysis and has its own homeostasis. In our model, the initial whole blood scaffold contains mainly fibrin, blood cells, and all other blood contents, and after degradation with fibrinolysis, they were replaced by collagen secreted by recruited cells, with further myofibroblast-associated smooth muscle differentiation. The cell source is sufficient *in vivo*, hence could avoid the problem of poor replicative capacity of high cell passages^37^ when culturing them *in vitro*. After 24 days of culture *in vivo*, there was no apparent fibrin remnant (Fig. 3) throughout the whole layer of the yielded tissue. It was known that fibrin degradation products could enhance collagen deposition when culturing vascular smooth muscle cells with fibrin scaffolds^36^, but further studies are needed to determine whether fibrin degradation products exert the same effect, and to what extent they do so, on the tissue-engineering myofibroblast *in vivo*.

### Mechanical stimulation

Mechanical stimulation in the bioreactor during fabricating TEBV was known to improve cell alignment^38^, mechanical properties^14,33,39–41^, smooth muscle differentiation^40^, contractility^41^, collagen component^14,33,39,40^, elastin component^41^ and vasoactive response^40^. We also observed enhanced mechanical properties and collagen content with increased frequencies of mechanical stimulation. However, the *in vitro* models have some major limitations, such as the shortening of TEBV during dynamic culture as compared to static culture^14,38^ and the increased circumferential alignment and mechanical properties at the expense of final length^33^. A study found that fibrin-based TEBVs with cyclic strain between 15–20% could cause permanent deformation and damages to the growing tissue and that up to 5 weeks of dynamic culturing was needed to confer TEBV better mechanical properties than static cultured controls^14^ which is a result similar to that of an earlier collagen-based TEBV model^41^. The cyclic strain in our model is 17.65% with the silicone tube of 2.5 mm outer diameter, which yielded tightly conformed tubular tissue without laxity or space between the tissues and the silicone tube when harvested.

*In vitro* mechanical stimulation of 3T3 fibroblasts in small intestinal submucosa extracellular matrix scaffold may increase the genetic expressions of collagen type I, aSMA, MMP-9, TGF-beta1 and TGF-beta3^42^. Another dynamic culturing of smooth muscle cells on small intestinal submucosa showed increases in collagen and elastin in the presence of VEGF or FGF-2, with the seeded small intestinal submucosa scaffold showing positive Verhoeff-van Gieson elastin fibrils staining^43^. There was an increasing trend in the amount of collagen quantification with increasing frequency of mechanical stimulation from 0.1 Hz to 0.5 Hz, this result is similar to our observations. However, the increased elastin was only found in the 0.1 Hz group, not in the 0.5 Hz group. But we observed increasing trends in both collagen and elastin associated with increasing frequency of mechanical stimulation, starting from the static, to the slow pumping (0.2 Hz), and then to the fast (0.5 Hz) pumping group. The difference might be related to the *in vivo* environment.

### *In vivo* culture

A major obstacle in previous attempts of using autologous subcutaneous tissues as vascular grafts is the tissue’s insufficient strength. While the tissue tube can be reinforced with polyester mesh^44^, this may lead to poor outcomes after it has been clinically implanted as arterial grafts^8^. Other approaches such as rolled-up^9^, layer-on-layer^11^, and addition of nicotine-coating^10^ have been introduced to increase the thickness of the tubular tissue and thus enhance mechanical properties. The addition of polyurethane sponge on the two ends^45,46^ is a method that has been applied for securing suture retention. Bregman *et al*.^12^ put pulsating polyurethane intra-aortic balloons, with 80 cycles/min, onto the dorsolateral subcutaneous area of dogs, and after 3 to 4 weeks the surrounding tubular tissue yielded had three times the tensile strength compared to that without pulsation. They used the tubular tissue as a graft of abdominal aorta in dogs and found that it remained patent even 6 to 8 months later. However, the boundary between the yielded tissue tube and the neighboring dermal tissue (unpulsed) was not clear^12^, making it complicated to evaluate and analyze the effect of cyclic mechanical stimulation.

A more recent study on sheep put a stretchable tubular device into the peritoneal cavity, exerting cyclic mechanical stimulation with 1 Hz and 20% strain for 8 days^47^. As in our study, using the peritoneal cavity instead of subcutaneous area avoided the interference of dermal fibroblasts and native skin ECM, and this approach also has the benefits of arresting minor infection in the subcutaneous segment spreading from the openings on the body surface, thus proving a larger space for the scaffold. After 10 days of implantation, however, the harvested tissue was not strong enough and ruptured after being grafted as a carotid artery interposition. The histology showed many porous spaces both in pulsed and unpulsed groups, and this finding was not observed in other studies with 2-week^16,17^ or 3-week^20^ peritoneal incubation, or in our study. ECM analyses showed no increases in collagen content, elastin content, or gene expression of elastin in the pulsed group, and the failure strength was less than 1/20 of the carotid artery. Furthermore, there was no observable elastin or aSMA expression in the pulsed or the unpulsed group. The shorter peritoneal incubation (10 days) might lead to the differences. In addition, with the 2-layer design, the outer elasteon sheath designed to avoid peritoneal adhesion might also avoid the biological contamination of the inner polyurethane tube that was mainly for the fabrication of the tissue tube. The minor immune provocation with minimal cell recruitment and associated collagen synthesis might thus make the yielded tissue frail.

### Limitations

One of the limitations of this study is the different length of the implanted silicone tubes between the static and pumping groups. Ideally the implanted devices should be the same between the static (control) group and pumping (study) group. But we chose shorter silicone tubes for the pumping group to reduce the injuries to the animals (rats) because they had wide dissections of the subcutaneous tissue on the back and trunk for implantation of the tube set and two openings left on the back, which cause extra injuries in comparison with the static group. The intraperitoneal portion of silicone tube of both the static and pumping groups went across the peritoneal cavity, being slightly bended to avoid distress of internal organs, but with both ends of silicone tube fixed on bilateral abdominal muscle walls for the static group and both ends of silicone tube extended into and fixed in the subcutaneous area for the pumping group. The impact on the yield of the tissue tube introduced by the difference in the sites of fixation of the silicone tube between the static and pumping groups should be minimal.

The overall mortalities in the first part surgery of LPS-whole blood scaffold implantation of about 10% were mainly observed in rats with less body weight or ill-appearance before the surgery. The deaths were presumably to be related to blood loss (from scaffold fabrication and during the whole surgery) or prolonged surgery. We did not perform cleaning or irrigation of the blood scaffold for removal of surface remnant LPS upon implantation, which should be improved in future studies. The dosage of LPS in our model was only 0.89% and 0.056% of known E.coli LPS LD_50_ (lethal dose, 50%) of aged and young mice (intraperitoneal injection, 1.6 mg/kg for aged and 25.6 mg/kg for young^48^), or 0.14% of LD_25_ (lethal dose, 25%) of rats (intraperitoneal injection, 10 mg/kg^49^). However, whether those deaths were directly related to the effects of LPS, and to what extent remains unclear. Nonetheless, aseptic techniques throughout the operation procedures are necessary, because infection of the scaffold will inevitably result in failure of the experiment.

The survival rate in the second part of surgery (aorta implantation) was approximately 1/3. Most of the deaths took place within 24 hours after the surgery and were most likely resulted from thrombus formation in the implanted graft, because we did not observe apparent endothelial markers in the tissue tubes incubated *in vivo* for 24 days, whether cyclically pressurized or not. However, almost all the rats that survived the second part of surgery lived till the end of the study, with the vascular differentiation of endothelial marker VWF (Fig. 6b), myosin heavy chain (Fig. 6i–k), elastic fiber (Fig. 6l–n) and full graft patency. The expressions of myosin heavy chain and elastic fiber in the 4-month graft in the fast pumping group (Fig. 6k,n) did not overtly exceed those in the slow pumping group (Fig. 6j,m), which was different from the findings right after peritoneal incubation (Figs 3n,o, 4h,i). The finding might be related to the fact that fast pumping produced a relatively “stiffer” vascular graft with relatively lower sustained mechanical strain up to 4 months as compared to slow pumping (Fig. 6o, 0.41 ± 0.06 vs. 0.57 ± 0.09, a 39% difference in the compliance ratios). Whereas more researches and quantifications are required for refining the strategy to fabricate the optimal vascular graft, the mechanical stimulation during the incubation period can lead to higher mechanical properties such as burst pressure and suture retention force (Fig. 1f), which are indispensable for safety concern as a vascular graft. The minimum and maximum implant-aorta ID ratios (Fig. 6o), which are indicators of luminal dilation of the graft that can be interpreted as structural stability, indicated smaller dilation in the fast pumping group compared to the slow pumping and control groups, although the differences did not reach statistical significance. Because structural stability is highly demanded for any clinical application, further large scaled studies are needed to confirm our speculation. In the analysis of ultrasonography images, we found layer-like structure depicted in Fig. 6a as horizontal lines. Similar image could be identified in the carotid ultrasonography measurement of intima-media thickness, where histology showed differences in density^50^, similar to those shown in our work (Fig. 6g); however the nature, genesis of this layer-like structure or its possible consequence regarding the vessel function remains to be investigated.

Future studies should be focused on refinements of the scaffold or coating on the implanted silicone tube in order to generate tissue tubes with better homogeneity, stability, and anti-coagulation property, so that they can serve as even better vascular grafts.

## Conclusion

We present a new *in vivo* tissue engineering model combining cell recruitment/activation, cyclic mechanical stimulation, and autologous whole blood scaffold to fabricate a vascular graft. The approach is different from those *in vitro* tissue engineering approaches that adjust the cytokines, culture medium, cell types, and other parameters. With cyclic mechanical stimulation during the culture period, the ECM (including collagen and elastin), smooth muscle differentiation, and mechanical properties can be enhanced by the end of incubation period in the peritoneal cavity. Although the application of the products is limited by the lack of initial endothelium and typical elastic structure of native large blood vessels, our model has the advantages of infinite cell sources, natural hormonal/cell-cell signal transduction, and normal homeostasis of the *in vivo* environment. Further studies are warranted to overcome those limitations, so that this product can meet clinical needs.

## Supplementary information

Representative gels of western blot analysis of tissue tubes incubated in the peritoneal cavity after 24 days

## Data Availability

The datasets generated during and/or analysed during the current study are available from the corresponding author on reasonable request.

## References

[1] Writing Group M (2016). Heart Disease and Stroke Statistics-2016 Update: A Report From the American Heart Association. Circulation.

[2] Di Mauro M (2005). Reoperative coronary artery bypass grafting: analysis of early and late outcomes. Ann Thorac Surg.

[3] Yap CH (2009). Contemporary Results Show Repeat Coronary Artery Bypass Grafting Remains a Risk Factor for Operative Mortality. Annals of Thoracic Surgery.

[4] Khot UN (2004). Radial artery bypass grafts have an increased occurrence of angiographically severe stenosis and occlusion compared with left internal mammary arteries and saphenous vein grafts. Circulation.

[5] Machiraju VR (2004). How to avoid problems in redo coronary artery bypass surgery. J Cardiac Surg.

[6] Desai M, Seifalian AM, Hamilton G (2011). Role of prosthetic conduits in coronary artery bypass grafting. European journal of cardio-thoracic surgery: official journal of the European Association for Cardio-thoracic Surgery.

[7] McAllister TN (2009). Effectiveness of haemodialysis access with an autologous tissue-engineered vascular graft: a multicentre cohort study. Lancet.

[8] Hallin RW, Sweetman WR (1976). The Sparks’ mandril graft. A seven year follow-up of mandril grafts placed by Charles H. Sparks and his associates. Am J Surg.

[9] Sakai O (2007). Development of the wing-attached rod for acceleration of “Biotube” vascular grafts fabrication *in vivo*. Journal of biomedical materials research. Part B, Applied biomaterials.

[10] Sakai O (2009). Faster and stronger vascular “Biotube” graft fabrication *in vivo* using a novel nicotine-containing mold. Journal of biomedical materials research. Part B, Applied biomaterials.

[11] Ma N (2011). Development of the novel biotube inserting technique for acceleration of thick-walled autologous tissue-engineered vascular grafts fabrication. Journal of materials science. Materials in medicine.

[12] Bregman D, Wolinsky H (1974). Autogenous vascular replacements induced by a subcutaneous pulsatile system. The Journal of surgical research.

[13] Seliktar D, Black RA, Vito RP, Nerem RM (2000). Dynamic mechanical conditioning of collagen-gel blood vessel constructs induces remodeling *in vitro*. Annals of biomedical engineering.

[14] Syedain ZH, Weinberg JS, Tranquillo RT (2008). Cyclic distension of fibrin-based tissue constructs: evidence of adaptation during growth of engineered connective tissue. Proceedings of the National Academy of Sciences of the United States of America.

[15] Syedain ZH, Tranquillo RT (2009). Controlled cyclic stretch bioreactor for tissue-engineered heart valves. Biomaterials.

[16] Campbell JH, Efendy JL, Campbell GR (1999). Novel vascular graft grown within recipient’s own peritoneal cavity. Circulation research.

[17] Gu GL (2010). Peritoneal cavity as bioreactor to grow autologous tubular urethral grafts in a rabbit model. World journal of urology.

[18] Chue WL (2004). Dog peritoneal and pleural cavities as bioreactors to grow autologous vascular grafts. Journal of vascular surgery.

[19] Campbell GR (2008). The peritoneal cavity as a bioreactor for tissue engineering visceral organs: bladder, uterus and vas deferens. Journal of tissue engineering and regenerative medicine.

[20] Zhang ZX (2008). *In vitro* study of endothelial cells lining vascular grafts grown within the recipient’s peritoneal cavity. Tissue engineering. Part A.

[21] Hauser J (2009). Enhanced cell adhesion to silicone implant material through plasma surface modification. Journal of materials science. Materials in medicine.

[22] Hu JJ, Chao WC, Lee PY, Huang CH (2012). Construction and characterization of an electrospun tubular scaffold for small-diameter tissue-engineered vascular grafts: a scaffold membrane approach. Journal of the mechanical behavior of biomedical materials.

[23] Dombros N (2005). European best practice guidelines for peritoneal dialysis. 3 Peritoneal access. Nephrol Dial Transplant.

[24] Leporatti S (2006). Elasticity and adhesion of resting and lipopolysaccharide-stimulated macrophages. FEBS Lett.

[25] Pi J (2014). Detection of lipopolysaccharide induced inflammatory responses in RAW264.7 macrophages using atomic force microscope. Micron.

[26] Patel NR (2012). Cell elasticity determines macrophage function. PloS one.

[27] Terada T (2011). Immunohistochemical profile of normal mesothelium and histiocytic/methothelial hyperplasia: a case report. Int J Clin Exp Pathol.

[28] Roh JD (2010). Tissue-engineered vascular grafts transform into mature blood vessels via an inflammation-mediated process of vascular remodeling. Proceedings of the National Academy of Sciences of the United States of America.

[29] Munford RS (2005). Detoxifying endotoxin: time, place and person. J Endotoxin Res.

[30] Kisch T (2015). LPS-Stimulated Human Skin-Derived Stem Cells Enhance Neo-Vascularization during Dermal Regeneration. PloS one.

[31] Ma Bin, Dohle Eva, Li Ming, Kirkpatrick Charles James (2015). TLR4 stimulation by LPS enhances angiogenesis in a co-culture system consisting of primary human osteoblasts and outgrowth endothelial cells. Journal of Tissue Engineering and Regenerative Medicine.

[32] Zeuner M, Bieback K, Widera D (2015). Controversial Role of Toll-like Receptor 4 in Adult Stem Cells. Stem Cell Rev.

[33] Syedain ZH, Meier LA, Bjork JW, Lee A, Tranquillo RT (2011). Implantable arterial grafts from human fibroblasts and fibrin using a multi-graft pulsed flow-stretch bioreactor with noninvasive strength monitoring. Biomaterials.

[34] Gui LQ (2014). Construction of Tissue-Engineered Small-Diameter Vascular Grafts in Fibrin Scaffolds in 30 Days. Tissue Eng Pt A.

[35] Syedain ZH, Tranquillo RT (2011). TGF-beta 1 diminishes collagen production during long-term cyclic stretching of engineered connective tissue: Implication of decreased ERK signaling. J Biomech.

[36] Ahmann KA, Weinbaum JS, Johnson SL, Tranquillo RT (2010). Fibrin degradation enhances vascular smooth muscle cell proliferation and matrix deposition in fibrin-based tissue constructs fabricated *in vitro*. Tissue engineering. Part A.

[37] Poh M (2005). Blood vessels engineered from human cells. Lancet.

[38] Weidenhamer NK, Tranquillo RT (2013). Influence of cyclic mechanical stretch and tissue constraints on cellular and collagen alignment in fibroblast-derived cell sheets. Tissue engineering. Part C, Methods.

[39] Solan A, Dahl SL, Niklason LE (2009). Effects of mechanical stretch on collagen and cross-linking in engineered blood vessels. Cell transplantation.

[40] Schutte SC, Chen ZZ, Brockbank KGM, Nerem RM (2010). Cyclic Strain Improves Strength and Function of a Collagen-Based Tissue-Engineered Vascular Media. Tissue Eng Pt A.

[41] Isenberg BC, Tranquillo RT (2003). Long-term cyclic distention enhances the mechanical properties of collagen-based media-equivalents. Annals of biomedical engineering.

[42] Gilbert TW (2007). Gene expression by fibroblasts seeded on small intestinal submucosa and subjected to cyclic stretching. Tissue engineering.

[43] Heise RL, Ivanova J, Parekh A, Sacks MS (2009). Generating elastin-rich small intestinal submucosa-based smooth muscle constructs utilizing exogenous growth factors and cyclic mechanical stimulation. Tissue engineering. Part A.

[44] Sparks CH (1973). Silicone mandril method for growing reinforced autogenous femoro-popliteal artery grafts *in situ*. Annals of surgery.

[45] Watanabe T, Kanda K, Ishibashi-Ueda H, Yaku H, Nakayama Y (2007). Development of biotube vascular grafts incorporating cuffs for easy implantation. Journal of artificial organs: the official journal of the Japanese Society for Artificial Organs.

[46] Watanabe T, Kanda K, Ishibashi-Ueda H, Yaku H, Nakayama Y (2010). Autologous small-caliber “biotube” vascular grafts with argatroban loading: a histomorphological examination after implantation to rabbits. Journal of biomedical materials research. Part B, Applied biomaterials.

[47] Stickler P (2010). Cyclically stretching developing tissue *in vivo* enhances mechanical strength and organization of vascular grafts. Acta biomaterialia.

[48] Tateda K, Matsumoto T, Miyazaki S, Yamaguchi K (1996). Lipopolysaccharide-induced lethality and cytokine production in aged mice. Infect Immun.

[49] Alexander HR (1991). Single-dose tumor necrosis factor protection against endotoxin-induced shock and tissue injury in rats. Infect Immun.

[50] Kim GH, Youn HJ (2017). Is Carotid Artery Ultrasound Still Useful Method for Evaluation of Atherosclerosis?. Korean Circulation Journal.

