# Supplementary material for: Combination of inductive effect of lipopolysaccharide and in situ mechanical conditioning for forming an autologous vascular graft in vivo
Source: Sci Rep. 2019 Jul 23;9:10616. doi: 10.1038/s41598-019-47054-2 (PMC6650437; doi:10.1038/s41598-019-47054-2)
Supplement: Supplementary file 1 — Representative gels of western blot analysis of tissue tubes incubated in the peritoneal cavity after 24 days [file 41598_2019_47054_MOESM1_ESM.pdf]

Article title:

Combination of inductive effect of lipopolysaccharide and in situ mechanical conditioning for forming an autologous vascular graft in vivo

Authors:

Chao-Lin Chen, How-Ran Guo, Ying-Jan Wang, Hong-Tai Chang, Chui-Yi Pan, Ho-Yi Tuan-Mu, Hsiu-Chuan Lin, Chao-Yi Chen, Jin-Jia Hu

Representative gels of western blot analysis of tissue tubes incubated in the peritoneal cavity after 24 days (from left to right: aorta, static, slow pumping, fast pumping)

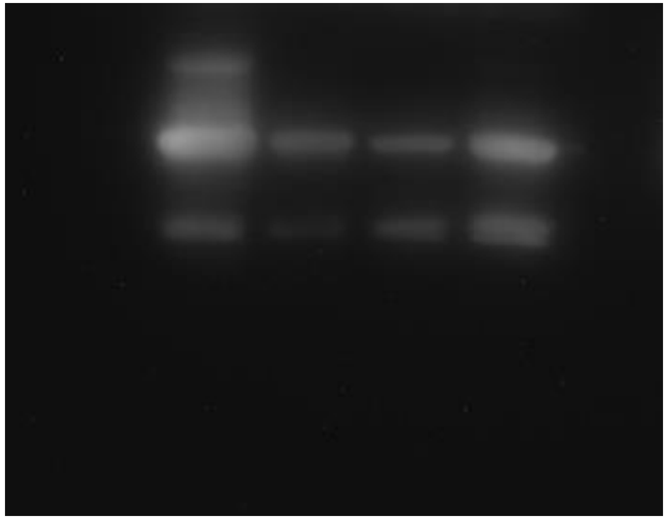

Alpha-SMA

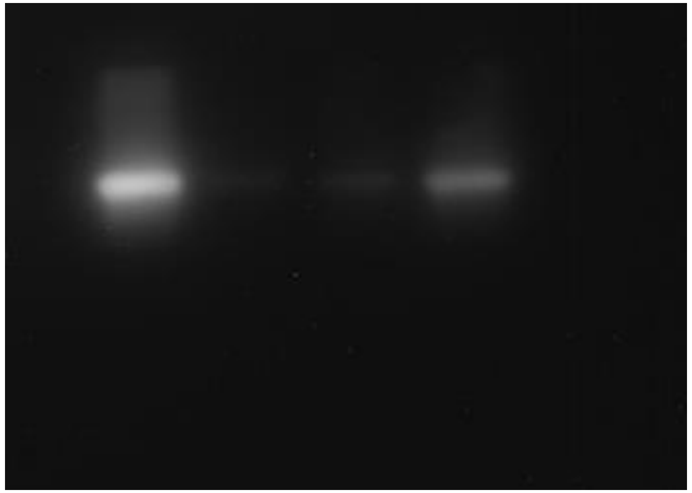

calponin

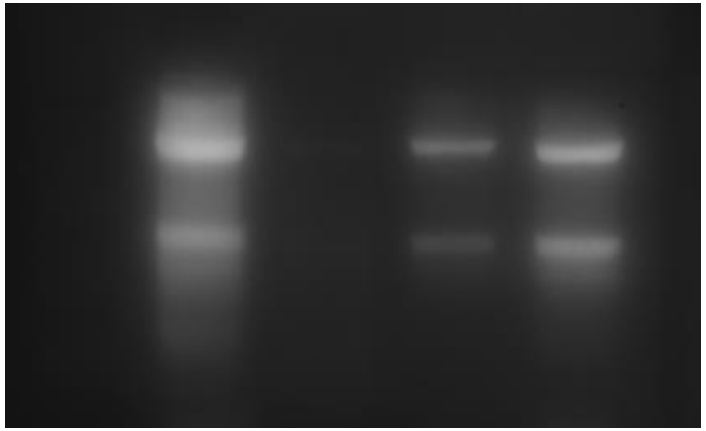

Myosin heavy chain
(BT-562)

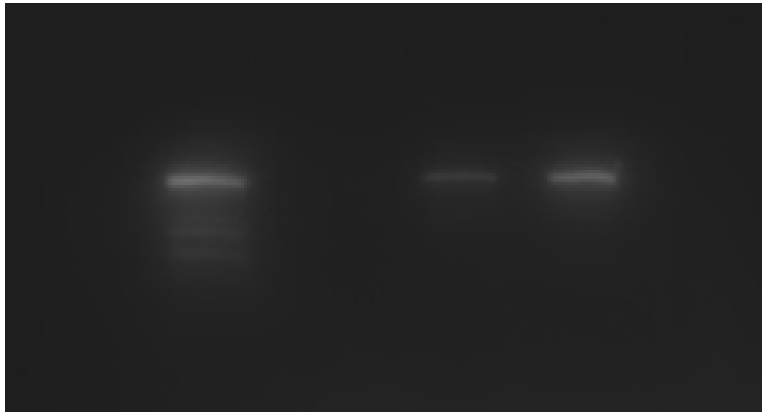

Myosin heavy chain
(ab683)

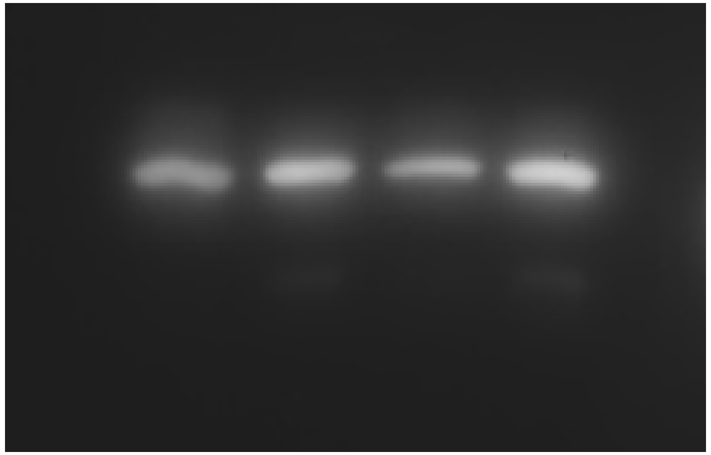

Beta-actin
